# Poly(lactide-*co*-glycolide)/Hydroxyapatite Porous Scaffold with Microchannels for Bone Regeneration

**DOI:** 10.3390/polym8060218

**Published:** 2016-06-07

**Authors:** Ning Zhang, Yang Wang, Wenpeng Xu, Yong Hu, Jianxun Ding

**Affiliations:** 1 Department of Foot and Ankle Surgery, The Second Hospital of Shandong University, Jinan 250033, China; zhangningno1@126.com (N.Z.); handfootsurgery@126.com (Y.W.); chinaowenxu@126.com (W.X.); 2 Changchun Institute of Applied Chemistry, Chinese Academy of Sciences, Changchun 130021, China

**Keywords:** poly(lactide-*co*-glycolide), hydroxyapatite, porous scaffold, microchannel, cell ingrowth, mass exchange, bone tissue engineering

## Abstract

Mass transfer restrictions of scaffolds are currently hindering the development of three-dimensional (3D), clinically viable, and tissue-engineered constructs. For this situation, a 3D poly(lactide-*co*-glycolide)/hydroxyapatite porous scaffold, which was very favorable for the transfer of nutrients to and waste products from the cells in the pores, was developed in this study. The 3D scaffold had an innovative structure, including macropores with diameters of 300–450 μm for cell ingrowth and microchannels with diameters of 2–4 μm for nutrition and waste exchange. The mechanical strength in wet state was strong enough to offer structural support. The typical structure was more beneficial for the attachment, proliferation, and differentiation of rabbit bone marrow mesenchymal stem cells (rBMSCs). The alkaline phosphatase (ALP) activity and calcium (Ca) deposition were evaluated on the differentiation of rBMSCs, and the results indicated that the microchannel structure was very favorable for differentiating rBMSCs into maturing osteoblasts. For repairing rabbit radius defects *in vivo*, there was rapid healing in the defects treated with the 3D porous scaffold with microchannels, where the bridging by a large bony callus was observed at 12 weeks post-surgery. Based on the results, the 3D porous scaffold with microchannels was a promising candidate for bone defect repair.

## 1. Introduction

Recently, orthopedic reconstruction procedures stemming from trauma, tumor, deformity, degeneration, and an aging population have dramatically increased, triggering a high demand on the improvement of bone implant technology [1,2]. Modern clinical practice in orthopedics has demonstrated that the autograft exhibits a superior therapeutic effect in bone fusion. However, donor site morbidity and limited supply are major concerns. Allografts and xenografts may raise other concerns in pathogen transmission and immunorejection, respectively [3,4]. Therefore, the development of synthetic materials for musculoskeletal tissue engineering is paramount in order to satisfy the booming demand of increased orthopedic implantations.

In the field of musculoskeletal tissue engineering, a plethora of biodegradable tissue substitutes have been created and tested, and some early products were marketed. Due to the complex structure required for three-dimensional (3D) thicker tissues [5], science is some way off from generating a definitive clinical product for bone tissue regeneration. This is largely due to the non-homogeneous growth of cells on the traditional ‘‘porous block’’ scaffolds, which prevent the formation of a functional construct from surface to core. It is due to the mass transfer limitations of the porous structures, into which cells are expected to invade and populate. The movement of nutrients to and waste products from the cells in the pores depends on molecular diffusion; nutrients are used up before reaching the inner core of the construct, and waste products build up. Cells that do migrate into the core become necrotic, and so the cell population is commonly found to be concentrated at the periphery of the scaffold [6].

Dunn *et al.* introduced an *in situ* solidification system composed of a poly(lactide-*co*-glycolide) (PLGA) implant in the 1990s [7]. This implant was dissolved in water-miscible solvents, such as *N*-methyl-2-pyrrolidone (NMP) and dimethyl sulfoxide (DMSO). The solid implants were formed *in situ* due to phase inversion triggered by solvent/non-solvent exchange. After contact with the aqueous medium, the high water miscibility of the solvents resulted in a fast phase inversion of the polymer solutions, and thus solidification of the system took place in seconds to minutes. Also, because of the phase inversion, a microchannel structure was created [8,9,10]. For example, Ellis *et al.* produced PLGA flat sheet membranes with a finger-like structure using NMP as a solvent and water as a non-solvent [6,11]. Porous structures are expected to form in the high mutual affinity of the NMP-water medium. Oh *et al.* fabricated the hydrophilic porous PLGA tubes using a modified immersion phase-inversion method and showed that the tubes were highly effective for the permeation of bovine serum albumin (BSA) [12]. These studies inspired us to devise a 3D porous scaffold with microchannels for bone repair *via* the phase inversion method to improve mass transport.

In this study, we fabricated an innovative 3D porous scaffold by phase inversion/particulate leaching method (PI), which possessed both macropores and microchannels, providing space for cell invasion and mass transfer, respectively. In our lab, the 3D porous scaffolds by melt-molding/particulate leaching method (MM) were studied for many years [13]. In order to explore the advantage of PI scaffold (S_PI_), the scaffold fabricated by MM (S_MM_) was applied to compare it in terms of structure, porosity, mechanical property, cell attachment, cell proliferation, osteogenic differentiation, and the capability of bone repair *in vivo*.

## 2. Materials and Methods

### 2.1. Preparation of PLGA/HA Homogeneous Hybrid Composite

The homogeneous hybrid composite composed of PLGA (Viscosity-average molecular weight (*M*_η_) = 147,000 Da, LA:GA = 75:25 (mol/mol), Changchun SinoBiomaterials Co., Ltd., Changchun, China) and hydroxyapatite (HA) (Nanjing Emperor Nano Material Co., Ltd., Nanjing, China) (HA:PLGA = 1:9 (*W*/*W*)) was prepared by the solvent-mixing method. Briefly, HA powder was uniformly suspended in 20-fold (by wt %) chloroform by magnetic stirring and ultrasonic treatment. The suspension was added into a 5% PLGA/chloroform solution to achieve the 10 wt % HA in the hybrid composite. The mixture was precipitated in an excess of ethanol, and the composite was dried in air for 48 h and vacuumed for 72 h to remove the residual solvent.

### 2.2. PLGA/HA Scaffold Fabrication via PI Method

A PLGA/HA scaffold was fabricated *via* combining phase inversion and particulate leaching (S_PI_). Firstly, two grams of PLGA/HA hybrid composite were added into 10.0 mL of NMP (Aladdin, Shanghai, China). After magnetic stirring under 70 °C for 6 h, the homogeneous PLGA/HA/NMP mixture was obtained. Next, the sieved sodium chloride particulates of 300–450 μm in diameter were added into the PLGA/HA/NMP mixture. The weight ratio of the salt particulates to PLGA/HA was 6:1. The mixture was cast in a homemade glass cylinder with a removable bottom. To remove NMP and salt particulates, the bottom was removed, and the mixture was immersed in distilled water for three days with the water exchanged every 12 h. Subsequently, any water remaining in the scaffold was exchanged by ethanol. Finally, the PLGA scaffold was obtained after three days of lyophilization and sterilized with ethylene oxide for 6 h. In addition, A PLGA/HA film was also fabricated. In detail, a drop of the PLGA/HA/NMP mixture were laid on the Φ8 mm round siliconized slide and then smeared on the surface homogeneously. Subsequently, the coated slides were immersed in 500.0 mL of distilled water for 10 min to remove NMP. The obtained PLGA/HA film-coated slide by phase inversion (F_PI_) would be used for cell culture in the next.

### 2.3. PLGA/HA Scaffold Fabrication via MM Method

A PLGA/HA scaffold was fabricated by a melt-molding/particulate leaching method (S_MM_). Briefly, the sieved sodium chloride particulates of 300–450 μm in diameter were added into the melted HA/PLGA hybrid composite in an internal mixer at 150 °C and 60 rpm. The weight ratio of salt particulates to the composite was 6:1. The obtained mixture was then molded into 3-mm-thick sheets under 10 MPa pressure at 150 °C for 5 min, and then cooled to room temperature. The salt particles were removed from the molds by leaching in distilled water for two weeks, and the water was changed every 12 h. Finally, the porous scaffolds were obtained after dried in air for 48 h and vacuumed for 72 h to remove the residual solvent, and sterilized with ethylene oxide for 6 h. In addition, a PLGA/HA film was also fabricated by melt-molding method (F_MM_) as process of F_PI_ fabrication.

### 2.4. Characterizations of Scaffolds

The porosities of scaffolds were determined using the ethanol replacement method. The microstructures of the scaffolds were examined by scanning electron microscopy (SEM; Philips XL30, Philips, Amsterdam, The Netherlands). The scaffolds were fractured after snap-freezing, sputter-coated with gold, and observed at an accelerating voltage of 15 kV. For characterizing the distribution and exposure degrees of HA in PLGA matrix, it was analyzed with energy dispersive X-ray spectrometry (EDX) (XL-30W/TMP, Philips, Amsterdam, The Netherlands). Rectangular bars of 30 mm × 5 mm × 5 mm in dry and wet state were chosen for mechanical strength tests measured by a universal testing machine (Instron 1121, Norwood, MA, USA). The compressive strength was measured at a crosshead speed of 2 mm/min. The stress histogram was obtained to determine mechanical properties. Three replicates were tested for the wet and dry conditions (*n* = 3).

### 2.5. Cell Culture

#### 2.5.1. Isolation of Rabbit Bone Marrow Mesenchymal Stem Cells (rBMSCs)

Three-month-old New Zealand white rabbits were selected for rBMSC isolation according to an established protocol. The animals were provided by Jilin University, Changchun, China, and treated according to the NIH Guide for the Care and Use of Laboratory Animals (NIH Publication No. 85-23, revised 1996). Bone marrow aspirates (5 mL) were obtained from rabbit tibia and subsequently cultured. Briefly, the isolated cell pellets were resuspended in 5.0 mL of culture medium (DMEM; Dulbecco’s Modified Eagle Medium (Gibco, Carlsbad, CA, USA) supplemented with 10% (*V*/*V*) fetal calf serum (Gibco, Carlsbad, CA, USA) and 100 IU/mL penicillin-streptomycin (Sigma, Shanghai, China)). The cells were seeded in culture dishes (Corning Costar Co., Cambridge, MA, USA) and cultured in a 37 °C and 5% carbon dioxide (CO_2_) incubator. Non-adherent cells were removed when the medium was changed after 24 h. After that, the medium was replaced every three days until the cells reached 80% confluence. Then, the cells were washed twice with phosphate-buffered saline (PBS), detached by treatment with 0.25% trypsin-ethylenediamine tetraacetic acid (EDTA, Sigma, Shanghai, China), and subcultured under the same condition until the third passage.

#### 2.5.2. Cell Adhesion

For adhesion studies, rBMSCs were seeded onto F_PI_, F_MM_, and glass at an initial seeding density of 1 × 10^4^ cells/cm^2^ and incubated for 4, 10, and 24 h. The cells were washed three times with phosphate-buffered saline (PBS), fixed with 4% (*W*/*V*) paraformaldehyde (PFA) at room temperature for 10 min, dyed with 2% fluorescein isothiocyanate (FITC) DMSO/H_2_O solution for 10 min, and then washed with PBS three times. Cell attachment was observed qualitatively under a reverse microscope (TE2000U, Nikon, Tokyo, Japan). The fluorescence pictures were taken by Digital Camera DXM1200F (Nikon, Tokyo, Japan) and analyzed with “NIH ImageJ” software (>15 per sample).

#### 2.5.3. Cell Proliferation

To investigate the cell proliferation on F_PI_, F_MM_, S_PI_, and S_MM_, rBMSCs were cultured on the two-dimensional (2D) films (F_PI_ and F_MM_) at a density of 1 × 10^4^ cells/cm^2^ and 3D scaffolds (S_PI_ and S_MM_) at a density of 2 × 10^4^ cells/cm^2^. After the indicated incubation times, the medium was replaced by Cell Counting Kit-8 (CCK-8, Dojindo, Japan). After 3 h of incubation, the absorbance values at 450 nm were measured on multifunction microplate scanner (Tecan Infinite M200, Männedorf, Zürich, Switzerland).

#### 2.5.4. Cell Differentiation

Alkaline phosphatase (ALP) activity was determined after culturing the cells in DMEM/F12, FBS (10%, *V*/*V*) for five and 10 days. Briefly, the medium of each well was carefully removed. Then, the cells were washed with PBS three times, lysed in radioimmunoprecipitation assay (RIPA) buffer, frozen at −80 °C for 30 min, and thawed at 37 °C. Then, *p*-nitrophenol phosphate substrate (*p*NPP) solution (Aladdin, Shanghai, China) was added, and the samples were incubated in the dark for 30 min at 37 °C. The reaction was terminated with 3.0 M sodium hydroxide (NaOH), and the ALP activity was read on a multifunction microplate scanner at 405 nm.

Calcium (Ca) deposition was determined by alizarin red S (ARS) staining of the rBMSCs after culture in DMEM/F12, FBS (10%, *V*/*V*) for 14 and 21 days. After three 5 min rinses in water, the scaffolds were incubated in ARS stain solution (0.1% ARS in Tris-HCl buffer, pH 8.0, Sigma-Aldrich,, St. Louis, MI, USA) for 30 min at 37 °C. The scaffolds were then washed in distilled water three times for 5 min each. The stained samples were treated with 10% (*W*/*V*) cetylpyridinium chloride in 10.0 mM sodium phosphate for 15 min at room temperature. The absorbance of ARS at 540 nm was recorded on a multifunction microplate scanner.

### 2.6. In Vivo Animal Study

#### 2.6.1. Implantation for Radius Defect Repair

Bilateral critically sized defects of nine rabbits were created with saw and drill in the radius of each rabbit forelimb by removing 2.0 cm of midshaft diaphyseal bone. A total of 18 radius defects were randomly divided into three groups with six defects treated with S_PI_ and S_MM_, respectively, and six defects in blank group as control.

The porous scaffold bars (0.3 cm in width, 2.0 cm in length) of S_PI_ and S_MM_ were placed into the defects of different rabbits, separately. The wounds were closed with silk threads in layers. After surgery, the rabbits were returned to their cages and allowed to move freely. All rabbits were injected daily with penicillin intramuscularly in a dose of 200,000 units for each one for three days. All the wounds healed gradually and the rabbits were active with no post-surgery complications.

#### 2.6.2. X-ray Examination

*In vivo* osteogenesis at the rabbit radius defects repaired with S_PI_ and S_MM_ were examined with Digital Radiograph (DR, Philips Digital diagnost, Philips, Amsterdam, The Netherlands) at baseline and 12 weeks post-surgery. The rabbits were exposed to X-ray in prone position during anesthetization. Afterwards, X-ray films were exported as TIF images. The newly formed bone was identified for quantifying its size and calculating its area fraction within the proportional area of its original bone defect region using a free and open ImageJ software developed by National Institutes of Health. All X-rays films were scored by the Lane-Sandhu scoring system [14].

### 2.7. Statistical Analyses

The data were presented as mean ± standard deviation (SD). The independent and replicated experiments were used to analyze the statistical variability of the data analyzed using Student’s *t*-test. *p* < 0.05 was considered to be statistically significant, and *p* < 0.01 and *p* < 0.001 were considered to be highly significant.

## 3. Results and Discussion

### 3.1. Scaffold Characterizations

**Figure 1:**
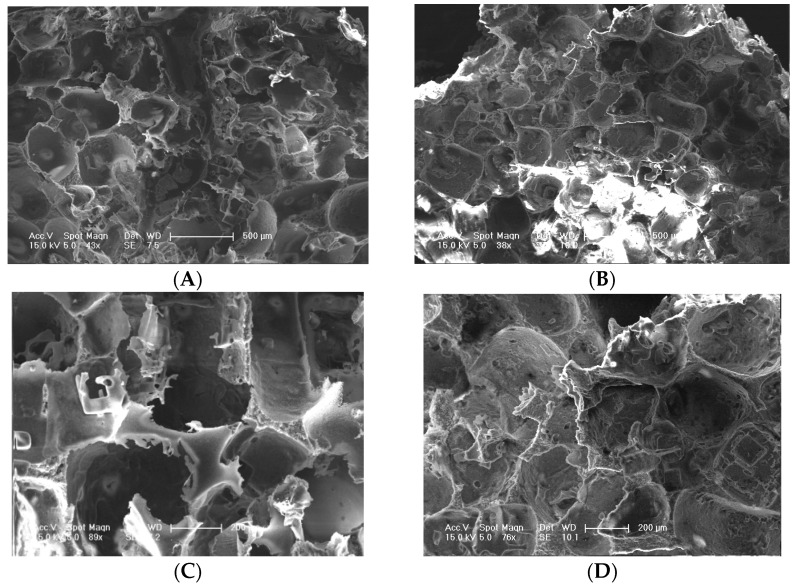
SEM images of S_PI_ (**A**,**C**) and S_MM_ microstructures (**B**,**D**). The bar lengths are 500 μm (**A**,**B**) and 200 μm (**C**,**D**), respectively.

**Figure 2:**
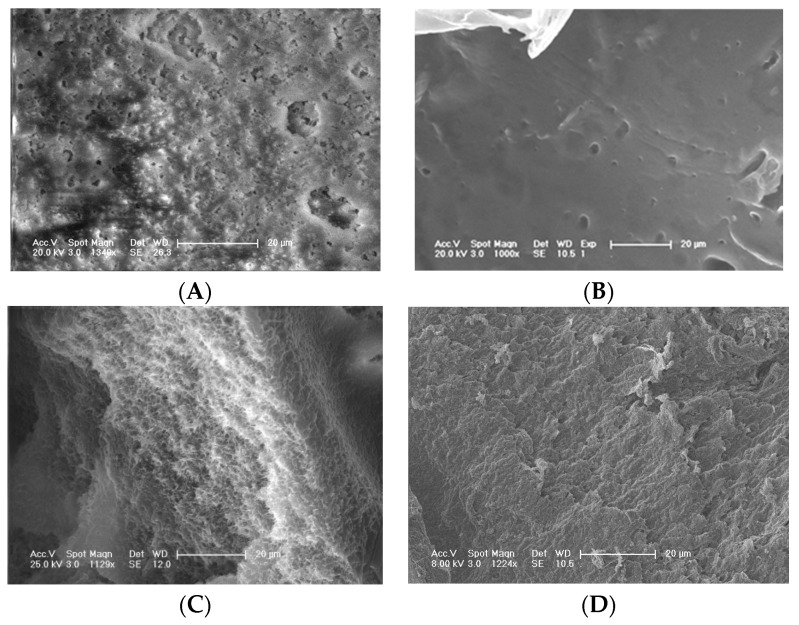
SEM images of pore surface and wall of S_PI_ (**A**,**C**) and S_MM_ (**B**,**D**). The bar length is 20 μm.

The microstructures of S_PI_ and S_MM_ were analyzed with SEM (Figure 1). Figure 1 shows that both fabricated scaffolds have the irregular macropores, and most of them are interconnected, which was suited for cell infiltration. The opening porosities of S_PI_ and S_MM_ are 87.27% ± 6.84% and 85.72% ± 9.45%, and there are no significant differences statistically (*p* > 0.05). However, the architecture of the pore surface and wall were different between them. As shown in Figure 2, the wall of S_PI_ had a honeycomb-like structure composed of microvoids with diameters of 2–4 μm, and the surface displayed microscale channels to which the internal macropores and microvoids in the skeleton were connected. The observed architecture was very favorable for the movement of proteins [12]. In contrast, the S_MM_ showed a smooth pore surface and solid pore wall, which impeded the material exchange.

The formation of microscale channels and microvoids in S_PI_ is based on the phase separation and gelation behavior of a PLGA/HA/NMP solution. When PLGA/HA/NMP solution comes into contact with a non-solvent, the influx of the non-solvent into the surface results in a process of phase separation, whereby the solution separates into a polymer-rich and polymer-lean phase [15]. Eventually, the concentration of the polymer in the polymer-rich phase becomes high enough for gelation to occur. This results in “freezing” of the two-phase structure, leading to microscale channels and microvoids.

**Figure 3:**
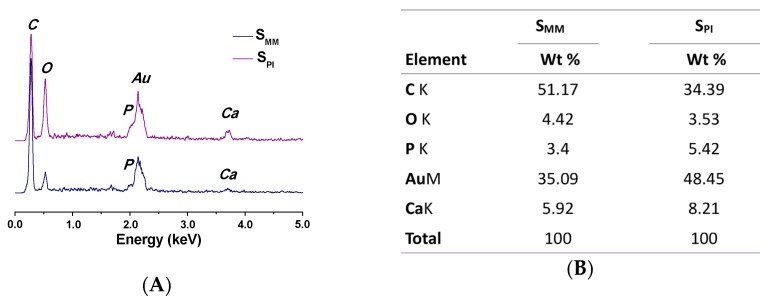
EDX spectra (**A**) and element contents (**B**) of S_PI_ and S_MM_.

EDX element analysis was used to assess the content of calcium (Ca), phosphorous (P), and oxygen (O) elements on the surface of the porous scaffolds. The EDX spectra of S_PI_ and S_MM_ are illustrated in Figure 3. The presence of HA in S_PI_ and S_MM_ was confirmed by the appearance of the characteristic peaks of Ca, P, and O, which are the main components of HA, while the Ca and P exposures in S_PI_ and S_MM_ were different. The weight percents (wt %) of Ca and P exposed on the surface of S_PI_ were 8.21 and 5.42 wt %, which were higher than those on the S_MM_ scaffold, *i.e.*, 5.92 and 3.4 wt %, respectively. This could be due to the microchannels in the surface, which increase the HA exposure.

**Figure 4:**
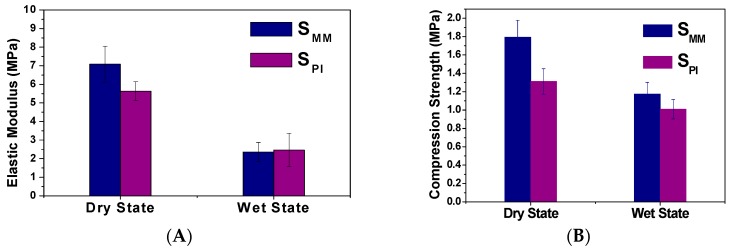
Elastic modulus (**A**) and compression strength (**B**) of S_MM_ and S_PI_ in dry and wet states.

The porous scaffolds are designed to provide mechanical support until the regenerative tissue or organ is structurally stabilized [16]. Therefore, the appropriate mechanical properties are crucial for such porous scaffolds. For instance, the fibrous scaffolds done by electrospinning also possess the interconnected structure for mass transferring, though they were rarely applied for bone defect repair in the weight-bearing area because of their poor mechanical property [17,18]. The scaffold should maintain its structural stability and integrity in an *in vivo* biomechanical environment and provide appropriate microstress stimulations for the implanted cells [19]. The mechanical property was often measured in the dry state. The compressive strength and elastic modulus were 1.31 ± 0.14 and 5.63 ± 0.51 MPa for S_PI_ and 1.80 ± 0.19 and 7.08 ± 0.97 MPa for S_MM_ (Figure 4), but they were different from that under physiological conditions, *i.e.*, in tissue fluid at 37 °C, because of the different media. As depicted in Figure 4, in wet state, the compressive strength and elastic modulus of S_PI_ reached 1.01 ± 0.11 and 2.46 ± 0.90 MPa, respectively, which is strong enough to offer structural support, and they are similar to those of S_MM_ (1.17 ± 0.13 and 2.37 ± 0.50 MPa) (Figure 4).

### 3.2. Cell Adhesion, Proliferation, and Differentiation

**Figure 5:**
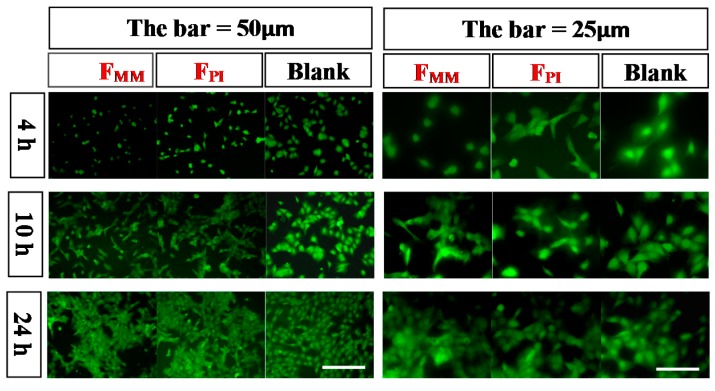
Cell adhesion on different substrates for 4, 10, and 24 h.

The behaviors of rBMSCs on F_PI_ and F_MM_ were investigated compared to a control substrate of glass. The cells were seeded and cultured for 4, 10, and 24 h on different substrates to assess cell adhesion (Figure 5). With the increase of culture time, the average area of cells on glass was highest, and the cells showed the best spread due to their good hydrophilicity. For F_PI_ and F_MM_, the cells also showed different behaviors. Cells on F_PI_ showed an elongated and spindle-like morphology after culture for 4 h and became a cuboidal morphology after 10 h. However, the cells on F_MM_ did not extend until culturing for 10 h. The average area of cells on F_PI_ was also much higher than that on F_MM_. It was believed that the roughness and topography of the pore surface increased direct cell-material binding, thus facilitating an increase in early cell adhesion [20,21]. After 24 h, the adhesive rates were similar between F_PI_ and F_MM_. The results suggested that the cell adhesion rate was related to the surface characteristics of materials, including stiffness, roughness, and hydrophilicity.

**Figure 6:**
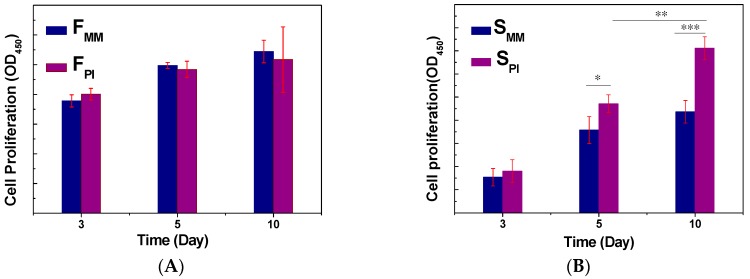
Cell proliferation on different films (**A**) and scaffolds (**B**) for three, five, and 10 days. Data are presented as mean ± SD (*n* = 6, *p < 0.05; **p < 0.01; ***p < 0.001).

The cell proliferation on the films (F_PI_ and F_MM_) and scaffolds (S_PI_ and S_MM_) was monitored quantitatively *via* CCK-8 assay to determine the metabolic activity of the total population of cells (Figure 6). After rBMSCs become attached to the surface, they enter a rapid proliferative growth phase in order to establish critical cell-cell interactions essential for the subsequent post-confluent differentiation growth phase. After culturing for three and five days on F_PI_ and F_MM_ films, rBMSCs showed significantly sustained growth. However, after 10 days of the cell seeding, the metabolic activity of the cells was not higher than that at five days in culture. It was deduced that the two-dimensional (2D) film cannot provide sufficient space for cell growth. In addition, there is no difference between F_PI_ and F_MM_ on cell proliferation during 10 days in culture. It means that the roughness of F_PI_ did not accelerate the growth of rBMSCs, which is not in accordance with the previous studies [20]. The cell proliferation on 3D scaffolds was different with that on 2D films. For S_MM_, the population of cells was significantly increased from Day 3 to 5 and increased slightly from Day 5 to 10, while the cells cultured on S_PI_ grew continuously from Day 3 to 10. Moreover, the metabolic activity of cells was higher than F_MM_ on Day 10. In brief, F_PI_ did not display better ability for cell growth compared to F_MM_, when rBMSCs were cultured on 2D films for 10 days. However, 3D S_PI_ improved the cell proliferation with respect to S_MM_. These results suggested that 3D S_MM_ lacks nutrition in the core of the scaffold after five days. Conversely, the special microstructure of S_PI_ was very favorable for the cell population in the core because the microchannels on the surface sped up the exchange of nutrients to and waste products from the cells in the inner scaffold.

**Figure 7:**
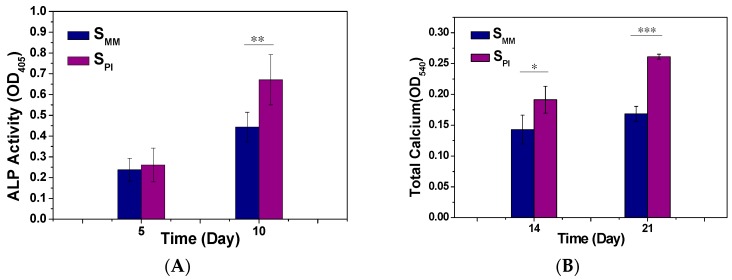
ALP activity (**A**) and Ca deposition (**B**) of S_MM_ and S_PI_. Data are presented as mean ± SD (*n* = 6, * *p* < 0.05; ** *p* < 0.01; *** *p* < 0.001).

The cell-biomaterial interactions have been demonstrated to exert a considerable influence on the differentiation and function of BMSCs [22]. To investigate the osteogenic differentiation of rBMSCs on S_PI_ and S_MM_, ALP activity and Ca deposition were measured. ALP is a membrane enzyme commonly recognized as a marker of osteoblastic differentiation. Figure 7A shows the ALP activities of the rBMSCs cultured on S_PI_ and S_MM_ after five and 10 days. The significantly higher ALP activity was detected in cells cultured on S_PI_ compared to S_MM_ on Day 10 (*p* < 0.05). Ca deposition was measured by ARS. As shown in Figure 7B, the quantification of ARS indicated that the deposition of Ca minerals in the S_PI_ was significantly higher than that in S_MM_ after culture for 14 and 21 days (*p* < 0.05). All results suggested that the cell differentiation toward osteogenesis was better on S_PI_ than S_MM_. It is well accepted that the surface structures, such as topographies, roughness, and nano-architectures, influence the cell and tissue behaviors of biomaterials [23,24]. So it is deduced that the stiffness and microchannels regulated cell proliferation.

### 3.3. Radius Bone Defect Repair

**Figure 8:**
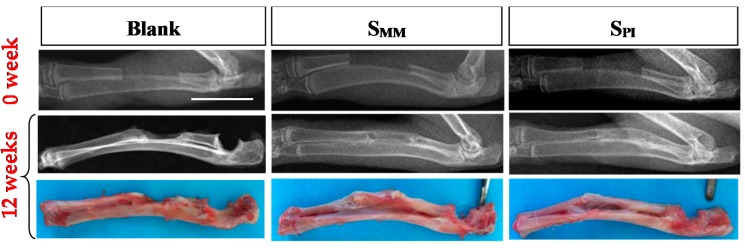
DR of rabbit radius defect at baseline; DR and macroscopic observation in Blank, S_MM_, and S_PI_ groups at 12 weeks post-surgery. The bar length = 20 mm.

**Figure 9:**
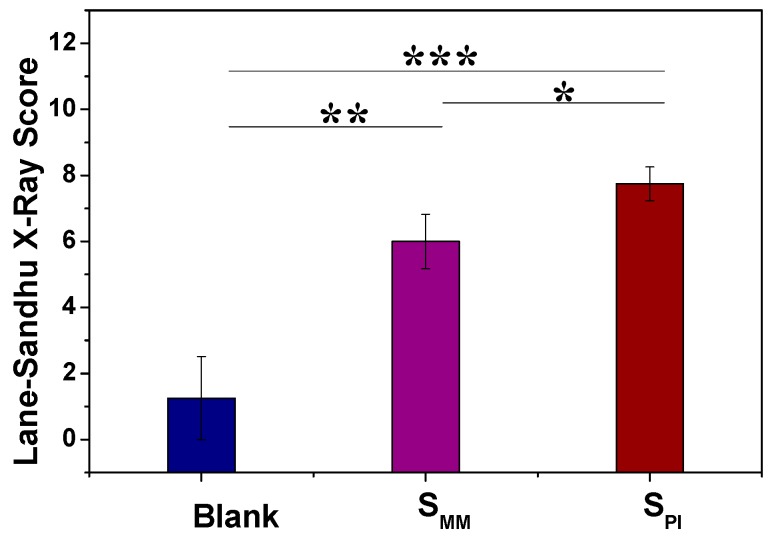
Lane-Sandhu X-ray scores in Blank, S_MM_, and S_PI_ groups at 12 weeks post-surgery. Data are presented as mean ± SD (*n* = 6, * *p* < 0.05; ** *p* < 0.01; *** *p* < 0.001).

At 12 weeks post-surgery, the defects without scaffold implantation are only filled with scattered bony structure (Figure 8). Bone defects implanted with S_MM_ revealed that the new bone tissue was concentrated at the periphery of the scaffold (Figure 8), which indicated that the osteocyte did not invade into the inner core of S_MM_ in the beginning, or the cells in the inner core were necrotic because of nutrition deficiency. In contrast, complete bridging between the bony ends is seen along the border of the radius after S_PI_ implantation (Figure 8). It is due to the countless microchannels across the inner scaffold, which was very favorable for substance exchange to avoid cell necrosis in the core. Quantitatively, the mean percentages of newly formed bone filling the segmental defect region were 17.21% for the defect group without scaffold implantation, 75.38% for S_MM_, and 92.22% for S_PI_, which is the highest among the three groups. The Lane-Sandhu X-ray scores of the three groups (Figure 9) after 12 weeks revealed that the S_PI_ group had the highest score and the blank group had the lowest one. The scores across the three groups were significantly different (*p* < 0.05). All results are due to the scaffold with microchannels that can improve cell attachment, proliferation, and differentiation.

## 4. Conclusions

The use of biomaterials to repair bone defects is a long and complicated process. This process depends on porosity and the ability to allow bone ingrowth [25]. In this study, a kind of porous scaffold with microscale channels was fabricated with phase inversion. The special structure was very favorable for the movement of nutrients to and waste products from the cells in the pores, which relies on molecular diffusion. Because of this, the cells can migrate and populate in the core of the scaffold. In addition, the stiffness and morphology of the pore surface facilitate cell attachment and cell differentiation. Therefore, the 3D porous scaffold with microchannels was a promising option for bone defect repair.
